# Smooth Sailing: A Pilot Study of an Online, School-Based, Mental Health Service for Depression and Anxiety

**DOI:** 10.3389/fpsyt.2019.00574

**Published:** 2019-08-20

**Authors:** Bridianne O’Dea, Catherine King, Mirjana Subotic-Kerry, Melinda Rose Achilles, Nicole Cockayne, Helen Christensen

**Affiliations:** ^1^Black Dog Institute, Prince of Wales Hospital, Sydney, NSW, Australia; ^2^Faculty of Medicine, University of New South Wales, Sydney, NSW, Australia

**Keywords:** school, student, mental health, stepped care, online, help-seeking, depression, anxiety

## Abstract

**Background**: Schools play an important role in supporting young people’s mental health, but face challenges identifying and responding to students in need of care. To assist secondary schools, the Black Dog Institute has developed an online, school-based, mental health service (Smooth Sailing). Delivered in the classroom, Smooth Sailing uses a website to screen, assess, allocate, and deliver care for depression and anxiety. The service is based on the principles of stepped care, offering treatments with varied intensity and follow-up by a school counselor when necessary. The current study aimed to evaluate the feasibility, acceptability, and safety of this new type of service among secondary school students.

**Methods**: Between February and June 2017, a single-arm, pre-post, pilot study was conducted among students from four NSW secondary schools. Schools were given access to the service for 6 weeks. Feasibility measures (consent rates and step allocations), acceptability measures (service use and satisfaction) and safety measures (deterioration in help-seeking intention scores and mental health symptoms) were assessed at baseline and completion of the 6-week trial period.

**Results**: A total of 59 students took part in the service pilot (mean age, 14.57 years; SD, 0.89 years; range, 13-16 years). At baseline, 18.64% of students were found to require follow-up from the school counselor, and 80% of these were new cases. Although completion of the online modules was low, service satisfaction was high. At 6 weeks, the mean scores for help-seeking, depression, and anxiety remained relatively stable or improved.

**Conclusions**: The current study presents important findings for the development and implementation of an online mental health service that screens students’ mental health and allocates care accordingly, all within the school setting. Although the findings provide some support for the feasibility, acceptability, and safety, service improvements are needed. The modifications outlined are likely to improve the quality of the service and its effectiveness.

**Trial Registration**: Australian New Zealand Clinical Trials Registry (ANZCTR):

ACTRN12617000977370

## Introduction

Given that half of all mental disorders emerge between the ages of 12 and 18 years (1), secondary schools play an important role in supporting the mental health of young people. Delivering mental health services in schools has the potential to address barriers to care, including accessibility, costs, and stigma (2). Many secondary schools employ counselors or psychologists to address student mental health, but up to one third report that their workload is unmanageable (3). A meta-analysis of face-to-face mental health services delivered to students found that targeted, selective, and universal programs were all effective for reducing mental health problems among students (4). Other initiatives, such as classroom-based online cognitive behavioral therapy (5–7) and curriculum-embedded mental health content (8) were also effective for improving symptoms and mental health literacy. However, wide-scale uptake is challenged by low levels of awareness of the effective mental health programs, competing priorities, time constraints, and limited resources (9). Despite the potential, schools remain underutilized, under resourced, and lack the capacity to appropriately manage students’ mental health needs.

Stepped care has been proposed as a service model for the treatment of depression and anxiety (10) that may increase engagement with care, reduce symptoms, and allow for better distribution of resources (11). Stepped care is considered well suited to depression and anxiety as these disorders are highly prevalent, have varied degrees of severity, are responsive to light-touch interventions, and the shortage of trained clinicians and specialist services hinders access to face-to-face care (12). Although there is no consistent definition of stepped care, it typically involves a process of screening and assessment to determine individuals’ symptoms and treatment needs (13). In some models, initial treatment is matched to the severity of symptoms whereas in others, all individuals begin at the same “step” of intervention, regardless of symptom severity. Individuals who fail to respond to their allocated treatment in the set time are then stepped up to the subsequent level of care (14). Most models do not incorporate stepping down. In accordance with clinical guidelines (15), stepped care for depression may involve several components including psychoeducation, self-directed online therapy, individual face-to-face therapy, medication, and monitoring. It has been argued that when fully realized, stepped care could maximize clinical outcomes while minimizing provider costs (13).

While the cost-effectiveness of stepped care has been supported (16–18), there is only emerging evidence of treatment effectiveness. A meta-analysis of 10 randomized trials among depressed adults found moderate treatment effects for stepped care, but limited evidence to support its use as the dominant treatment model (19). In a review of primary care trials, stepped care for depression was found to be as effective as treatment as usual (20); however, its clinical superiority was undetermined. Few formal evaluations of stepped care have been conducted among youth. When comparing standard care to stepped care for the treatment of clinical anxiety, no significant difference in symptom reduction was found (17). In contrast, young adults living with HIV who received stepped care for depression had significantly greater improvements in symptoms compared to those receiving treatment as usual (21). This is consistent with Mufson et al. (22) who found stepped care to be more effective for reducing depression among adolescents in primary care when compared to treatment-as-usual. This suggests that the superiority of stepped care may be dependent on the degree of intervention provided by the treatment comparator. As such, stepped care may be ideal for school settings where treatment as usual is minimal.

In Australia and other high-income countries, most schools operate on a wait-to-act model in which school staff instigate referral to mental health support only after observing certain behaviors or students’ self-disclosures (23). As help-seeking is low among youth, and teachers are not always trained to identify those in need (24), a proactive model like stepped care which detects symptoms and stratifies care accordingly may assist schools in caring for students. Components of stepped care have already been implemented in schools with some success. School based screening has been found to identify a significantly greater proportion of students to be in need of mental health services than would have been identified without screening (25). School based screening has also resulted in increased rates of referral, improved help-seeking behavior, and greater access to services for students (26). Australian schools are well-placed to provide professional follow-up due to the availability and employment of school counselors and school psychologists. Although other components of stepped care, such as triage and brief intervention, have been tested in school settings with promising results (27), it remains unknown whether Australian schools have the capacity to integrate a stepped care model for depression and anxiety. Given the emergence of mental illness in adolescence, the low levels of help-seeking, and limited school resources, evaluating stepped care and its promise is timely.

### The Smooth Sailing Service

The Black Dog Institute has developed an online, schools-based, mental health service called Smooth Sailing. Based on the principles of stepped care, Smooth Sailing uses a website to screen, assess, allocate, and deliver psychological interventions to improve help-seeking for mental health problems and reduce depressive and anxiety symptoms among secondary school youth. Brief, validated, self-report measures of depression and anxiety (28, 29) are used to accurately determine students’ symptoms and required level of care. The service has three degrees of treatment intensity which are matched to students’ initial symptom severity categorization (i.e. nil-mild, moderate, moderately severe to severe). The model is consistent with Australian Clinical Practice Guidelines (30) and conservative due to the novelty of the service. Self-directed, web-based, psycho-education is provided for students with nil to mild symptoms. Self-directed, web-based, cognitive behavioral therapy (CBT) is provided for students with moderate symptoms (5, 7). A direct link to face-to-face care with a school counselor is provided for students with moderately severe to severe symptoms or thoughts of death/harming one’s self. School counselors are instructed to provide their usual care, consistent with school guidelines and policies. Students’ symptoms are monitored fortnightly by an email or SMS check-in, which also includes a reminder to use the service and an automated login link. Every 6 weeks, students complete a step assessment from which care is reallocated based on their results.

**Figure 1:**
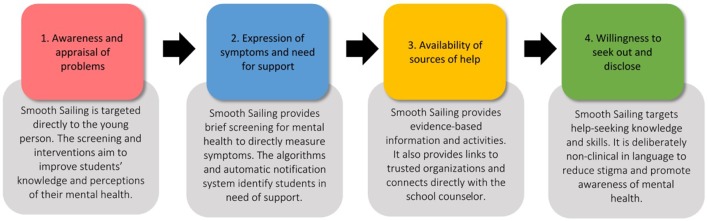
Rickwood et al.’s (31) help-seeking model applied to the Smooth Sailing service.

The Smooth Sailing service is based on Rickwood et al. (31) help-seeking model. As outlined in **Figure 1**, Smooth Sailing directly targets each of the stages of help seeking through varied content and features. Smooth Sailing utilizes the Internet to address resource shortages and provide young people with evidence-based information and resources (32, 33). A major strength of the current service is that it links directly to face-to-face care, improving the likelihood of actual and future help-seeking (34).

The Smooth Sailing service was designed in partnership with students, school counselors (3), teachers, General Practitioners (GPs; 35), and parents (36). These stakeholders strongly endorsed the service due to perceptions of its usefulness in detecting symptoms and providing care, the suitability of the school setting for reaching youth, and young people’s preferences for digital technology. However, key concerns also emerged including the privacy and confidentiality of students’ information, Internet accessibility, and the availability of face-to-face care. School counselors felt students may try to avoid follow-up by answering the screening questions dishonestly and that students may forget or lack motivation to complete the online modules. School staff felt that gaining parental consent would be a potential barrier, although conversely, parents endorsed the service due to the prevalence and impacts of poor mental health among youth. While these concerns have been raised in similar studies of school-based computerized programs for mental health (37–41), broad uptake and successful implementation of the service is unlikely unless issues related to feasibility and acceptability are addressed.

### Aims

The current study aimed to evaluate the feasibility, acceptability, and safety of the Smooth Sailing service among secondary school students. Feasibility was determined by the willingness of students to take part and the capacity of school counselors to initiate and manage follow-ups. Acceptability was determined by the extent to which students used the service, barriers to service use, and students’ perceptions of service satisfaction (42, 43). The safety profile of the service was determined based on the deterioration in students’ help-seeking intentions and symptoms after using the service. Although the study was not powered to detect significance (44, 45), measuring the change in these scores determined whether the service was likely to have an effect in an appropriately powered trial. The current study enabled service improvements to be defined and actioned, including important resource considerations. It also provided the initial data needed to develop future trial protocols, including sample size calculations and recruitment targets. Given the limited number of studies on school-based mental health services, and the lack of evaluation of stepped care for adolescent mental health, the current pilot may help researchers and clinicians to design more effective and integrative school-based service models.

## Method

### Study Design

A single-arm, pre-post, 6-week uncontrolled pilot trial was conducted. The study was approved by UNSW Human Research Ethics Committee (#167424), the NSW State Education Research Application Process (#2016471), and the necessary Catholic Education Offices. The study was undertaken in NSW, Australia, between February and June 2017. The recruitment target was set at a minimum of 50 students in total from 4 schools. This target was based on recommendations for pilot research (46, 47) and was conservative to minimize the number of notifications and potential overburden on the participating school counselors. A convenience sample of schools who had expressed interest in the service during the design phase was used. School principals were emailed an information letter inviting the school counselor and two class groups from each school to participate. For school consent, schools were asked to provide a signed letter of support from the school principal. This letter was then forwarded to the governing ethics bodies to confirm school participation. Upon receipt of the signed school letter of support, student information and consent forms were mailed to each school. These forms were then distributed to the selected class groups by school staff. Interested students were required to return their consent form with signed parental consent by the day of the first school researcher visit. There were no other exclusion criteria. The study information sheet and consent form informed the students that taking part was completely voluntary, and that they were free to withdraw from the study at any time, without penalty, and without having to give a reason. Students could withdraw by emailing the research team or notifying the researchers at the school visits. Parents could also withdraw their child at any time using the same methods or by contacting their child’s school.

### Implementing the Service

At baseline, researchers visited the school to deliver the service in class time. Researchers reviewed students’ consent forms and provided them with instructions to register to the service. Registration involved visiting the service website (https://smoothsailing.blackdoghealth.org.au) and completing an online Gillick Competency measure—six multiple choice questions to test students’ capacity to provide informed consent and their understanding of the service. During registration, students provided their name, study code, email, mobile phone number, gender, and date of birth. They were asked to report their current employment status (part-time/casual, nil), whether they identified as lesbian, gay, bisexual, trans, or intersex (LGBTI) (answered yes, no, I’d rather not say) or as Aboriginal or Torres Strait Islander (ATSI) (answered yes, no, I’d rather not say). They were also asked to report whether they knew someone with a mental illness; cared for someone with a mental illness; had a mental illness themselves; or had used the Internet to find information about a mental health problem (all answered yes, no). This information was collected to determine the demographics of the sample alongside their experience and exposure to mental health problems.

The self-report mental health screener consisted of two validated measures: the nine-item Patient Health Questionnaire (PHQ-9; 28) for depressive symptoms and the seven-item self-report Generalized Anxiety Disorder Scale (GAD-7; 29) for generalized anxiety symptoms. Each of these questionnaires listed symptoms, and students were asked to rate how frequently they had experienced these, in the past 2 weeks, using a four-point Likert scale ranging from not at all (0) to nearly every day (4). The service automatically calculated a total score for each scale. Using whichever total score was the highest, students’ symptoms were classified as “nil-mild” (i.e. total score on PHQ-9 or GAD-7 equaling 0-9), “moderate” (i.e., total score of PHQ-9 or GAD-7 between 10 and 14), or “moderately severe to severe” (i.e., total score of PHQ-9 or GAD-7 between 15 and 27). To measure the impact of their symptoms on overall functioning, one additional item asked students to rate how difficult their symptoms had made their daily life and relationships. Participants answered using a four-point Likert scale ranging from not at all (0) to very (4).

After completing the screener, the service automatically allocated students to a step of care that matched their symptom severity (see **Table 1**). The Smooth Sailing service produced a personalized dashboard which provided students with an overview of the recommended modules to complete in their own time (see **Table 2**). The online psycho-education consisted of five 10-minute modules which provided information about anxiety, depression, and help-seeking. The modules were created specifically for the Smooth Sailing service and were reviewed in the co-design process by young people as well as a clinical psychologist. The content was also edited by a copywriter to ensure it was written at an appropriate reading level. The modules are complemented by animations and illustrations as well as hyperlinks to other credible youth mental health services and websites. All modules are designed to be self-directed, self-paced, and can be completed in any order. Module 6 includes referral to two web-based, publicly available, free, evidence-based CBT programs for depression and anxiety (5, 48, 49). MoodGym (5) comprises of five modules in which young people learn strategies to identify and manage unhelpful patterns of thinking, connect their thoughts and feelings, improve self-esteem and interpersonal relationships, and relaxation exercises to de-stress. The BRAVE Program (48, 49) includes ten 1-hour self-directed sessions that are usually completed over 10 weeks, that teach young people to identify anxiety and stress, develop relaxation and problem-solving skills, and reframe negative thinking. Before ending the visit, researchers advised students to use the website as much as they wished for the next 6 weeks.

**Table 1:** Smooth Sailing Model: Criteria for steps and intervention provided.

	Step 0 to 1	Step 2	Steps 3 to 4
Total scores on PHQ-9 or GAD-7	0-9	10-14	15+
Symptom severity	Nil-Mild	Moderate	Moderately-severe to Severe
Self-directed online psycho-education	Yes	Yes	Yes
Self-directed online cognitive behavioral therapy (CBT)	No	Yes	Yes
Face-to-face session with School Counselor	No	No	Yes

**Table 2:** Overview of the Smooth Sailing modules.

Title	Content overview
What is mental health?	Information about mental health issues common among youth and when it might be time to seek help.
Feeling on edge	Information on anxiety, how to identify it, potential causes, where to seek help and practical tips for managing it.
Waves of sadness	Information on depression, differences between sadness and depression, potential causes, how and where to seek help, and practical tips to cope.
When it’s time to tell someone	Information about when to seek help, how to talk to friends and parents, seek help from a GP, and the roles of different health professionals.
When a mate needs a hand	Ways to help others including having a private chat, seeking help together, respecting the treatment process, and the importance of looking after yourself.
Don’t fret, help is here	This module offers access to two evidence-based free online CBT programs, produced by Australian universities. Young people can select which program they prefer. This module is only offered to those at steps 2 and above.

Students who were allocated to steps 3 and 4 (i.e. moderately severe to severe symptoms) and/or reported thoughts of that they would be better off dead or of harming themselves in the past two weeks (i.e. score ≥ 1 on item-9 of the PHQ-9) automatically triggered a notification to the school counselor for follow-up through a secure, deidentified, email. Using the study ID codes, this email outlined that a student required follow-up from the school counselor within two days. School counselors were provided with a paper list of student names and matching study ID codes to ensure follow-up could be facilitated. The screening was only conducted on days when the school counselors were onsite. After conducting the student session, researchers met with the school counselor to review the email notifications. This took no more than 30 minutes. School counselors were provided with a list of local mental health services to support their follow-up. Two days after the school visit, the research team contacted the school counselor to confirm that all students had been followed up and to monitor any adverse events. This procedure was repeated at 6 weeks. All data were collected *via* the Smooth Sailing service e-platform which is hosted on university servers at the Black Dog Institute, University of New South Wales, Sydney, Australia.

### Outcome Measures

**Table 3** presents the key outcome measures and the criterion used to determine whether service modifications and improvements to procedure were needed.

**Table 3:** Outcome measures.

Domain	Construct	Measured by	Criteria for service modifications
Feasibility	Willingness of students to take part	Percentage of students who gained parental consent	<50%
Feasibility	The school counselor workload	Percentage of students that triggered a follow-up notification	>20%
Acceptability	Service use	Percentage of students who were “minimal users” i.e. completed ≤ one module	>50%
Acceptability	Service satisfaction	Percentage of students who withdrew from the service Percentage of students who reported barriers to service use Percentage of students who agreed with the service satisfaction items	>20% >20% <60%
Safety	Incidence	Percentage of identified cases that were new i.e. students who reported current symptoms but did not have a prior history of mental illness	<50%
Safety	Likely effectiveness	Help-seeking scores and mental health symptoms at baseline and 6 weeks	Deterioration at 6-weeks

### Feasibility

*Consent rate:* This was measured by the percentage of students who gained parental consent to participate and determined the likelihood of service uptake among students. The service was deemed feasible if >50% of invited students took part, based on the uptake of previous Australian school-based mental health programs (50, 51). Non-consenting students were asked to complete a short anonymous questionnaire outlining their reasons and to indicate whether an incentive would encourage participation.

*Step allocations and follow-up notifications:* This was the percentage of students who were allocated to each step and the percentage who required follow-up from the school counselor. This was measured to provide an estimate of the prevalence of mental health issues among students and the capacity required of school counselors. Based on prior recommendations for school-based screening, the service was deemed feasible if no more than 20% of students triggered a follow-up notification (52).

### Acceptability

*Service use:* Based on a recent review of uptake and use of digital health interventions (53), service modifications were needed if the majority of students (>50%) were minimal users (i.e. completed one or less of the prescribed modules).

*Barriers to service use:* Service barriers were identified using an 18-item list delivered at 6-weeks. Students were asked to report whether they experienced any of the listed service barriers throughout the pilot (e.g. “I forgot how to access Smooth Sailing,” answered yes or no). If >20% of students reported experiencing the same barrier, service modifications were needed.

*Service satisfaction:* Satisfaction was measured using service dropout - the percentage of students who withdrew from the service throughout the pilot period. Based on dropout rates for mental health treatment, service modifications were needed if dropout was >20% (54). Satisfaction was also measured using students’ responses to an 11-item questionnaire delivered at 6 weeks. Students were asked to agree or disagree with a list of statements about the service (e.g. “Smooth Sailing was easy to understand”). Service modifications were needed if <60% of students agreed with each of the statements.

### Safety

*Incidence:* This was the number of new cases identified by the service i.e. the percentage of students who required follow-up but had no history of mental health problems or illness. Service modifications would be needed if <50% of the identified cases were new.

*Likely effectiveness:* This was determined based on the deterioration in help seeking and symptom scores at 6 weeks. Help-seeking intentions for mental health were assessed at baseline and 6 weeks using an adapted version of the General Attitudes to Help-Seeking Questionnaire (GHSQ; 55). Students were asked to rate how likely they were to seek help when faced with a mental health problem from 12 different sources including informal (e.g. parents, friends, other adults), formal (e.g. GP, mental health professional), school-based (e.g. teacher, school counselor), and technology (e.g. mental health websites, telephone helplines, Internet activities). Each item was answered using a 5-point scale ranging from “extremely unlikely’’ to “extremely likely.” Answers were summed to create a total score with higher scores indicating a greater likelihood of seeking help. As outlined, the service also measured students’ depression and anxiety symptoms at baseline and 6 weeks using the Patient Health Questionnaire (PHQ-9; 28) and the Generalized Anxiety Disorder Scale (GAD-7; 29). Higher scores indicated greater symptom severity.

## Results

### Feasibility

**Figure 2:**
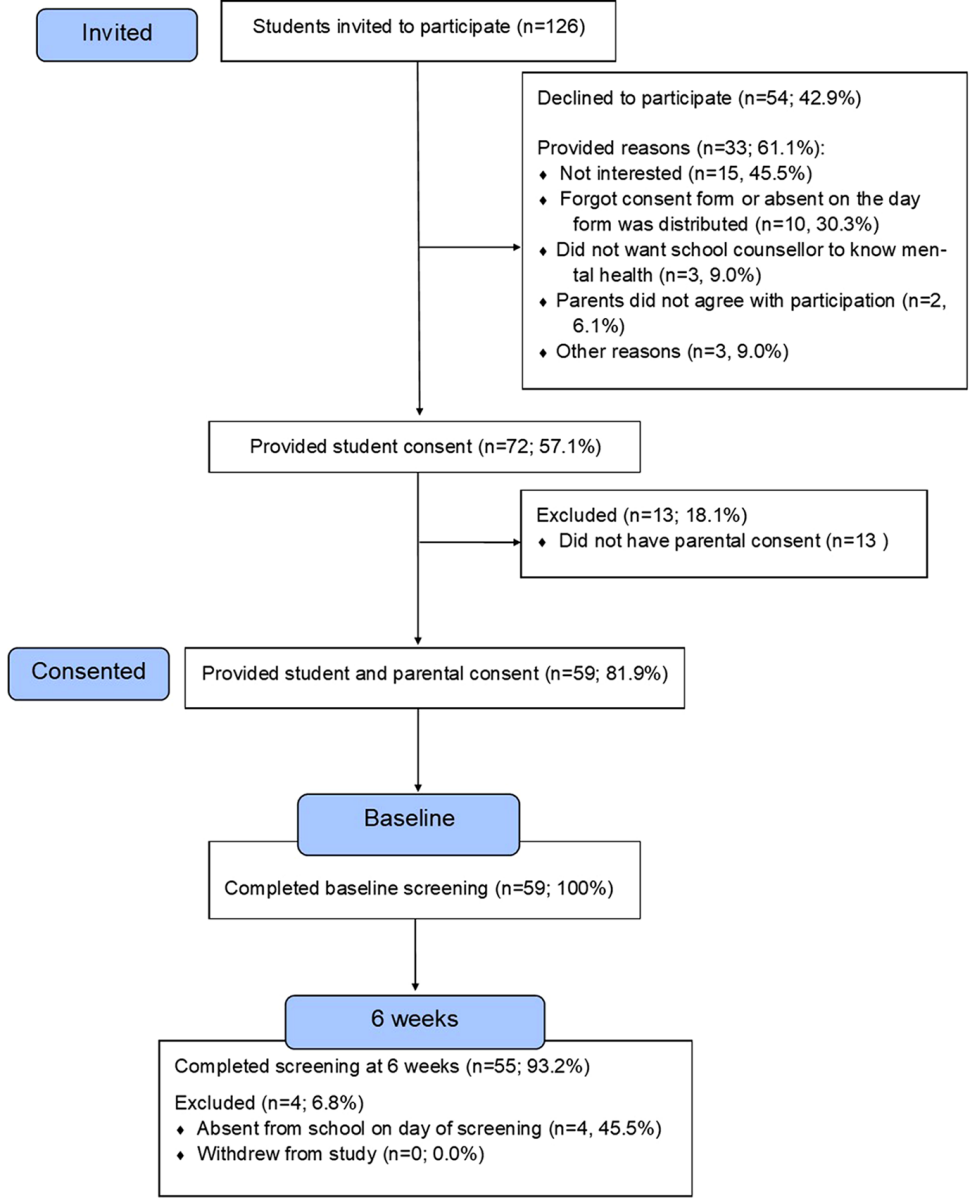
The CONSORT flowchart outlining recruitment and service participation.

*Consent rate:* **Figure 2** outlines the participant recruitment and flow.

A total of 126 students from the 4 participating schools were invited to take part in the service and 72 provided their consent (57.14%, *n* = 72/126). Of these, 59 gained parental permission. This gave an overall consent rate of 46.83% (*n* = 59/126). A total of 33 non-consenting students completed the feedback form. Of these, half were male (*n* = 18/33, 54.54%) and over half reported feeling sad, worried, or stressed for more than two weeks at a time (54.54%, *n* = 18/33). **Figure 2** outlines the reasons for non-participation. When asked about the use of an incentive for future participation, 24.24% (*n* = 8/33) said they would not participate regardless, 39.39% (*n* = 13/33) preferred a gift voucher, and 36.36% (*n* = 12/33) gave no response. Participant characteristics of the final sample are presented in **Table 4**, stratified according to baseline step allocation.

**Table 4:** Participant characteristics at baseline stratified by step allocation (N = 59).

	Steps 0 to 1 (n = 39)		Step 2 (n = 10)		Steps 3 to 4 (n = 10)	
	n	%	n	%	n	%
Female	23	58.97	6	60.00	6	60.00
Employed	7	17.95	3	30.00	0	0.00
Provided mobile number	20	51.28	7	70.00	3	30.00
Lesbian, gay, bisexual, trans, queer, intersex	1	2.56	0	0.00	1	10.00
Aboriginal or Torres Strait Islander	6	15.38	1	10.00	0	0.00
Knew someone with a mental illness	21	53.85	9	90.00	9	90.00
Cared for someone with a mental illness	13	33.33	8	80.00	5	50.00
Reported having a mental illness	6	15.38	1	10.00	3	30.00
Used Internet to find information about a mental health problem	5	12.82	2	20.00	5	50.00

**Figure 3:**
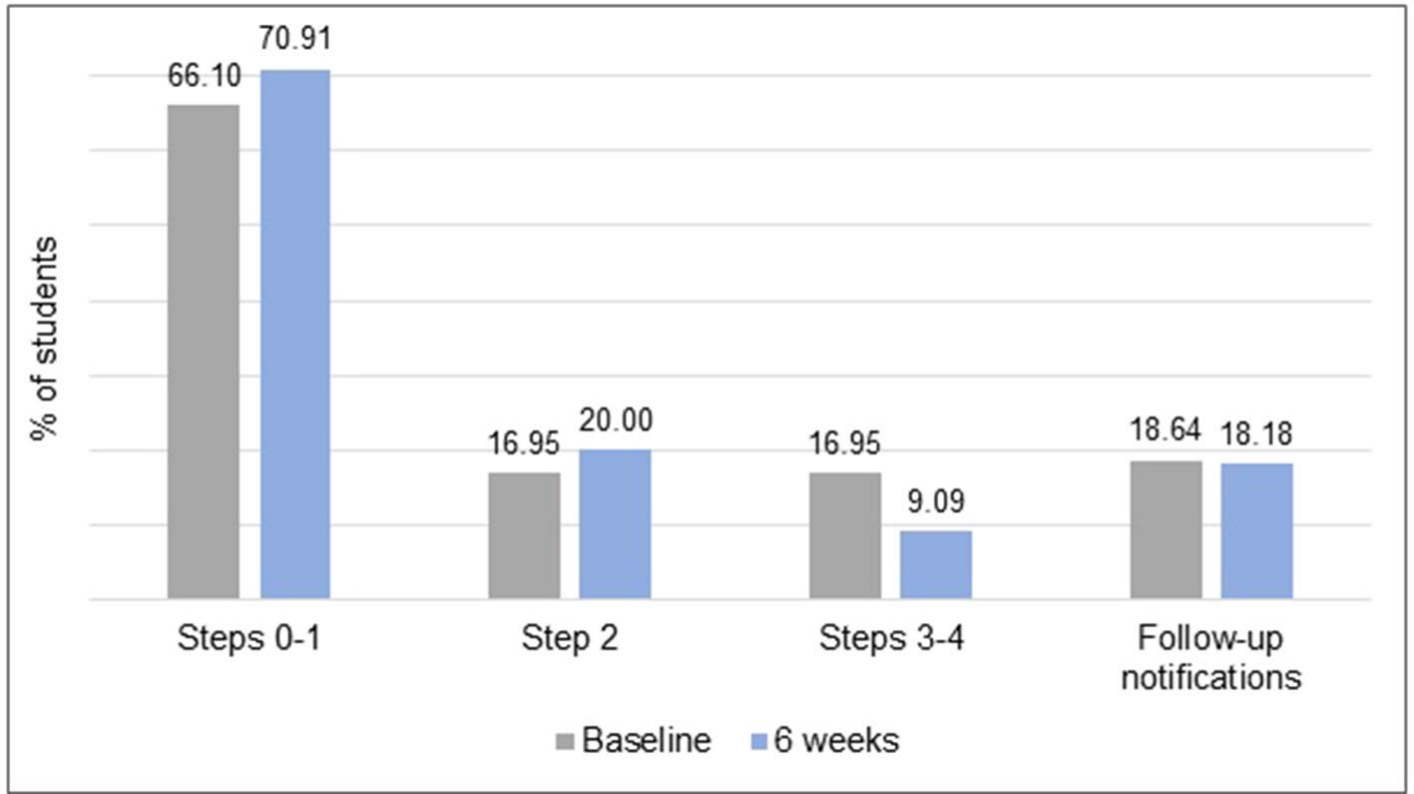
The frequencies of the step allocations and follow-up notifications at baseline and 6 weeks.

*Step allocations and follow-up notifications:* Displayed in **Figure 3**, two thirds (66.10%, *n* = 39) of the sample reported nil to mild symptoms at baseline and were allocated to steps 0 and 1; 16.94% (*n* = 10) reported moderate symptoms and were allocated to step 2; and a further 16.94% (*n* = 10) reported moderately severe to severe symptoms and were allocated to steps 3 and 4. A total of 18.64% of the sample (*n* = 11/59, 7 female) triggered a follow-up notification at baseline. There was a downward shift in symptom severity at 6-weeks with reductions in the number of students at the highest steps. However, there was no change in the total number of students who required follow-up at 6 weeks because five students with nil-mild symptoms reported thoughts of death and/or self-harm.

### Acceptability

*Service use:* Although module completion was higher among the students with more severe symptoms at baseline, the majority were minimal users (range: 50.00-74.35%, see **Table 5**).

**Table 5:** Service use among students throughout the pilot (N = 59).

	Steps 0 to 1 (*n* = 39)		Step 2 (*n* = 10)		Steps 3 to 4 (*n* = 10)	
	n	%	n	%	n	%
Nil modules completed	16	41.02	2	20.00	2	20.00
Only 1 module completed	13	33.33	4	40.00	3	30.00
2 or more modules completed	10	25.64	4	40.00	5	50.00

*Barriers to service use:* Outlined in **Table 6**, service barriers differed according to baseline step allocation. Students at step 2 experienced more barriers than others, reporting problems with Internet connectivity, lack of time, forgetfulness, low motivation, worry about the privacy of data, content taking too long to read and complete, using too much phone data, not trusting the service, feeling too worried or down to use the service, and not wanting the school counselor to know their feelings.

**Table 6:** Barriers to service use reported at 6 weeks (N = 55).

	Step 0 to 1 (*n* = 37)	Step 2 (*n* = 9)	Step 3 to 4 (*n* = 9)
My Internet connection didn’t work	Yes	Yes	NA
I didn’t have time to use Smooth Sailing	Yes	Yes	Yes
I forgot about Smooth Sailing	Yes	Yes	Yes
I couldn’t be bothered using Smooth Sailing	Yes	Yes	Yes
Smooth Sailing wasn’t what I needed	Yes	Yes	Yes
I forgot how to access Smooth Sailing	Yes	Yes	No
I was worried about privacy of my data	No	Yes	No
I didn’t want school counselor to know my feelings	No	Yes	Yes
I had trouble logging into the website	Yes	Yes	No
I felt too worried or down to use Smooth Sailing	No	Yes	No
The check-ins took too long to complete	No	Yes	No
Smooth Sailing took too long to read	No	Yes	No
Smooth Sailing used too much phone data	No	Yes	No
I didn’t trust Smooth Sailing	No	Yes	No
The text was too hard to read on phone	No	No	No
Smooth Sailing took too long to load	No	No	No
I didn’t have a phone or computer to use	No	No	No
Smooth Sailing made me feel worse	No	No	No

Yes indicates that the barrier was experienced by >20% of students and that service modifications are needed. No indicates that the barrier was experienced by <20% of students.

*Service satisfaction:* There were no active withdrawals during the service pilot and 55 of the 59 students were present for the 6-week assessment (93.22% retention). As outlined in **Table 7**, most of the students (range: 60–100%) felt that Smooth Sailing was easy to understand, easy to use, interesting and enjoyable, regardless of their baseline step allocation. The majority also felt comfortable providing their mobile phone number, agreed that they would tell a friend to use the service, and would use the service again in the future. The students allocated to steps 3 and 4 were comfortable with school counselor follow-up but less comfortable providing their email address. There was also disparity in students’ responses to whether Smooth sailing helped them “feel in control of their feelings” and “helped them a lot in everyday life,” with lower levels of agreeance among those allocated to the higher steps.

**Table 7:** Service satisfaction reported at 6-weeks (N = 55).

	Steps 0 to 1 (*n* = 37)	Step 2 (*n* = 9)	Steps 3 to 4 (*n* = 9)
Smooth Sailing was easy to understand	Yes	Yes	Yes
I found Smooth Sailing easy to use	Yes	Yes	Yes
I enjoyed using Smooth Sailing	Yes	Yes	Yes
I would tell a friend to use Smooth Sailing	Yes	Yes	Yes
I thought Smooth Sailing was interesting	Yes	Yes	Yes
I felt comfortable providing my mobile phone number	Yes	Yes	Yes
I would use Smooth Sailing again in the future	Yes	Yes	Yes
I felt comfortable providing my email address	Yes	Yes	No
I was comfortable being followed-up by the school counselor	Yes	No	Yes
Smooth Sailing helped me to feel in control of my feelings	Yes	No	No
The skills I learned helped me a lot in everyday life	No	No	No

Yes indicates that >60% of students agreed. No indicates that >60% disagreed and that service modifications are needed.

### Safety

*Incidence:* Sixteen of the 20 (80%) students who required follow-up did not report a history of mental health problems or mental illness.

*Likely effectiveness:* The mean scores for help-seeking intentions, depression, and anxiety appeared stable or improved throughout the pilot, across all levels of symptom severity (see **Table 8**).

**Table 8:** Help-seeking intentions (GHSQ), depression (PHQ-9), and anxiety (GAD-7) scores at baseline and 6 weeks (N = 55).

	Steps 0 to 1 (n = 37)		Step 2 (n = 9)		Steps 3 to 4 (n = 9)	
	Baseline	6 weeks	Baseline	6 weeks	Baseline	6 weeks
	M (SD)	M (SD)	M (SD)	M (SD)	M (SD)	M (SD)
Help-seeking intentions (GHSQ)	34.36 (6.88)	35.38 (7.74)	29.30 (11.07)	36.00 (9.67)	24.60 (6.50)	24.22 (0.36)
Depression (PHQ-9)	2.82 (2.59)	2.59 (3.27)	10.30 (2.63)	10.80 (3.33)	15.10 (3.38)	11.22 (5.26)
Anxiety (GAD-7)	2.36 (2.50)	2.20 (2.72)	9.60 (3.53)	10.00 (4.03)	12.70 (5.96)	9.44 (7.21)

## Discussion

This study aimed to determine the feasibility, acceptability, and initial safety profile of an online mental health service for improving help seeking and mental health symptoms in NSW secondary school students. The findings revealed that some modifications to the service and its procedure are needed if a future controlled trial is to be successful.

### Feasibility

Uptake of the service among students was low with less than half consenting to take part. Importantly however, the main barrier to consent appeared to be administrative: one quarter of the non-participating students had forgotten their consent forms and others were absent on the day forms were distributed. A more streamlined approach such as using passive “opt out” consent for parents and collecting student consent on the day of registration may improve uptake. This is likely to be supported by school communities as only a small proportion of parents did not want their child to participate in the service, although this would need to be evaluated further. Other strategies to increase uptake of the service among students may include broader promotion and marketing, utilizing school champions (56), increasing teacher awareness and support, and using incentives (57, 58). To better understand reasons for non-participation, future trials would benefit from implementing a consent form which all students are required to return, regardless of whether they choose to participate. This would allow more accurate rates of uptake to be measured and to better capture the reasons for non-participation. This is particularly important to address any concerns students have about using the service, the potential follow-up from the school counselor, and privacy protection.

This pilot also confirmed that the feasibility of the service is significantly impacted by the availability of school counselors to conduct the student follow-ups. In the initial screening, the service found that nearly one in four students experienced symptoms of depression and anxiety that warranted being seen by the school counselor. These rates are likely to increase the workload of school counselors. Based on the current study, school counselors would be required to initiate consultations with approximately 20% of all students screened. This has implications for delivering the service to larger samples, which would be needed for an effectiveness trial. In preparation for service implementation, schools would need to increase school counseling resources during the screening periods to ensure that all students requiring follow-up are seen in a timely manner. This would allay the concerns that students would not have access to face-to-face care if needed (3, 36). The service may lead to fewer follow-ups in the future; however, this would need to be investigated. Implementing the service over a longer period would enable researchers to evaluate the preventative and early intervention effects of the proposed stepped care model. As school counselors have already reported feeling time poor and vulnerable to burnout (3), future studies would need to monitor the impact of the increased workload on school counselors’ well-being and job stress.

### Acceptability

There was no drop-out throughout the pilot, signifying the support for this type of service among the participating youth. However, service use was low with most students failing to complete more than one module. This is problematic, particularly for the students allocated to step 2 (i.e. moderate symptoms) as they were symptomatic but failed to engage with the therapeutic content. Students at step 2 also faced more barriers to service use, such as poor Internet connectivity, failure to remember passwords, and forgetfulness. Time constraints also impacted students’ use. Although email and SMS reminders were used, these did not appear to increase engagement. While SMS reminders are more likely than emails to be actioned (59), only half of the students chose to provide their mobile phone number. Service use may be improved by schools allocating class time for module completion as Neil et al. (60) found this resulted in a threefold increase. Other strategies could include publishing promotional material throughout the schools, inserting a web link to the Smooth Sailing service on schools’ websites and students’ desktops, introducing multiple options for restoring access and password retrieval (e.g. one-time pin codes, email verification links, use of secret questions) and utilizing student leaders to promote the service. A future trial may also benefit from using multi-modal methods of reminders including ones that are classroom-based, as well as customizable electronic reminders *via* SMS and email. Highlighting the brief time commitment required to work through the online modules may also increase module completion. Engagement is a challenge for many Internet programs and interventions, with the relationship between adherence and effect still unclear (61). However, as greater adherence can lead to stronger effects (62), modifications to the service may be necessary to increase acceptability and effectiveness.

Service satisfaction was high. Most students reported that they enjoyed using the service and found it easy to use. The online delivery mode may have contributed to this, with young people commonly reporting positive experiences with Internet activities for mental health (63). However, for stepped care models to be effective, participants need to be motivated and engaged with their allocated treatment (64, 65). The findings suggest that the current service model may need to be modified to include additional support or interaction, particularly for those at step 2 because motivation and capacity to engage with the self-directed content was low. This confirms school counselors’ prior concerns that symptomatic students may have difficulties engaging with this type of delivery (3). In studies among adults, adherence to online therapies has been greater when external support or guidance was provided (66). The current service model may be improved by the addition of human contact for youth allocated to step 2. Further, many students reported that the service was not what they needed, despite many having symptoms. Students’ lack of awareness of their mental health may have negatively impacted their engagement with the service. This is supported by Gould et al. (67) who found that students’ perceptions about their need for treatment impacted their service use. The service may benefit from improving the registration process to better educate students about its purpose and include symptom feedback to help students understand their needs. Providing more information about the effectiveness of e-mental health programs to students (68) and utilizing parents or peers for support (69) may help increase service use and satisfaction. In addition, redefining the expectations of students who have nil-minimal symptoms and implementing a curriculum-embedded mental health program (8) may help to supplement the online modules and increase overall acceptability.

### Safety

A key question of this study was to establish the initial safety profile of a service which overtly identified students in need, provided care, and referred them to the school counselor when appropriate. The service was successful at detecting new cases of mental health problems, with four of five of the students identified not having a history of mental illness. Prior concerns about Internet-delivered screening and programs for school students have been related to them being untruthful or joking with their responses (3, 36). This study found no evidence of this behavior in students. On the contrary, it appeared that the service was perceived as trustworthy, encouraging honest disclosures of mental health history, symptoms, and help-seeking behavior. However, future studies would benefit from measuring the outcomes of student follow-ups, to determine whether the positively identified cases were genuine and whether referrals to other mental health services were made. The mean scores at baseline and 6 weeks suggested that the service did not worsen students’ intentions or symptoms, and positive significance may be found with a larger sample. Importantly, most students were comfortable being followed up by the school counselor, and even those who were not remained in the service. These findings provide some initial support for the effectiveness of the proposed model, but the low rates of module completion suggest improvements are needed before effects can be confirmed in a larger clinical trial. Future studies would benefit from increasing the sample size, using a control group, and implementing the service over a longer period. This would help determine genuine improvements in help-seeking behavior and symptoms.

## Conclusion

The current study presents important findings for the development and implementation of an online mental health service that screens students’ mental health and allocates care accordingly, all within the school setting. Although the findings provide some support for the feasibility, acceptability, and safety, service improvements are needed. Modifying consent procedures, ensuring school counselor availability, improving completion of modules, and removing service barriers related to accessibility will significantly improve the quality of the service and its likely effectiveness. The current study confirms the potential of this new type of service model for identifying new cases of mental health problems in students, which may halt symptom progression and prevent the onset of serious mental illness. A randomized controlled trial comparing this service to school as usual would determine the genuine effects and benefits for students. Future studies should also examine the impact of the service on school counselors, school culture, and parents. This would help to understand the broader implications of this new type of service delivery model.

## Ethics Statement

This study was carried out in accordance with the recommendations of the National Health and Medical Research Council's National Statement on Human Research with written informed consent from all subjects in accordance with the Declaration of Helsinki. The protocol was approved by UNSW Human Research Ethics Committee (#167424), the NSW State Education Research Application Process (#2016471), and the necessary Catholic Education Offices. Written consent was obtained from participants and their parents

## Author Contributions

BO’D and HC conceived the study. BO’D prepared the protocol and initiated the trial. HC and NC approved protocol and supervised trial activities. BO’D, CK, and MS-K contributed to the coordination of the trial. BO’D and CK contributed to data analysis. BO’D led the authorship of the paper alongside all co-authors. MA completed formatting and referencing. All authors read and approved the final manuscript.

## Funding

This project was funded by Hong Kong and Shanghai Banking Corporation (HSBC) and the Graf Family Foundation.

## Conflict of Interest Statement

The authors declare that the research was conducted in the absence of any commercial or financial relationships that could be construed as a potential conflict of interest.

## Abbreviations

(CBT), Cognitive Behavioral Therapy; (PHQ-9), Patient Health Questionnaire-9; (GAD-7), Generalized Anxiety Disorder 7-item; (UNSW), University of New South Wales; (GHSQ), General Attitudes to Help-Seeking Questionnaire; (AHSQ), Actual Help-Seeking Questionnaire; (LGBTI), Lesbian, Gay, Bisexual, Transsexual or Intersex; (ATSI), Aboriginal and Torres Strait Islander.
